# Autistic Traits and Gender Incongruence: Clinical perspectives from specialized psychiatric centres in Italy

**DOI:** 10.1192/j.eurpsy.2026.10464

**Published:** 2026-07-31

**Authors:** S. Leo, E. Zuliani, C. Gesi, A. C. Omboni, G. Gallo, F. Riva, L. Pasquarelli, B. M. Dell’Osso

**Affiliations:** 1 Department of Biomedical and Clinical Sciences “Luigi Sacco”, University of Milan, Milano; 2 Department of Mental Health and Addiction, ASST Brianza, Vimercate; 3 Department of Mental Health and Addiction; 4 Gender Clinic, ASST Fatebenefratelli-Sacco, Milano; 5 Gender Clinic, ASST Brianza, Vimercate, Italy; 6 MSc Data Science and Analytics, Alumnus, Brunel University, London, United Kingdom; 7 ”Sportello Trans” Gender Clinic, ALA Milano Onlus, Milano, Italy; 8 Department of Psychiatry and Behavioral Sciences, Stanford Medical School, Stanford, United States; 9 CRC “Aldo Ravelli” for Neurotechnology & Experimental Brain Therapeutics; 10 Centro per lo studio dei meccanismi molecolari alla base delle patologie neuro-psico-geriatriche, University of Milan, Milano, Italy

## Abstract

**Introduction:**

Scientific evidence is increasingly supporting the existence of a link between gender dysphoria (GD), or gender incongruence (GI), and autism spectrum disorder (ASD). It has been shown that transgender individuals, on average, present higher rates of ASD and ASD traits compared to cisgender individuals, which may complicate the diagnostic process for GD/GI and reduce access to care. Thus, this population may require a tailored clinical approach. However, the impact of ASD and ASD traits on GD/GI manifestations remains poorly understood.

**Objectives:**

The study aims to assess the co-occurrence of ASD and GD/GI symptoms and to explore their mutual relationship among transgender individuals and other cisgender groups.

**Methods:**

Adults (age 18-59) with a diagnosis of GI/GD and/or ASD were enrolled. A group of healthy subjects were also recruited through social media. All participants filled a set of self-rating scales, including Utrecht Gender Dysphoria Scale (UGDS-DS), Body Uneasiness Test (BUT), Camouflaging Autistic Traits Questionnaire (CAT-Q), Autism Spectrum Quotient (AQ) and Empathy Quotient (EQ). Clinical and socio-demographical data were also obtained through clinicians’ interviews. The overall sample was divided in four groups: 1) cisgender autistic individuals (ASD group, n=31), 2) transgender individuals without ASD traits (AQ<29 and EQ>30) (GD group, n=35), 3) transgender individuals presenting ASD traits (AQ>29 and EQ <31) or ASD diagnosis (GD+ASD group, n=22), 4) healthy subjects (CTL group, n=71).

**Results:**

Nearly half of transgender individuals (38.6%) presented ASD traits or diagnosis. They were older at GD/GI diagnosis, underwent less gender affirming procedures, had higher masking behaviours and suffered more from depressive symptoms compared to GD group. Group comparison by means of ANOVA are provided in table 1. Across the overall sample, AQ (B=5.44, p=0.015) and Global Severity Index of BUT scale (B=3.11, p<0.001) were predictive of higher UGDS-DS scores.

**Table 1.** Main effect of groups on psychometric scales scores. Table 1. Long description.

	**ASD** (mean±sd)	**GD** (mean±sd)	**GD+ASD** (mean±sd)	**CTL** (mean±sd)	**Tot** N=159 (mean±sd)	** Games-Howel post-hoc comparisons (p<0.05)**
**UGDS-DS ** n.miss.=0	39.96±16.17	77.00±11.46	78.22±8.09	32.63±7.12	50.14±23.17	GD, GD+ASD> ASD> CTL
**GSI (BUT index)** n.miss.=7	0.83±0.84	1.78±0.80	2.21±0.90	0.86±0.72	1.23±0.94	GD, GD+ASD> ASD, CTL
**AQ** n.miss.=1	33.97±6.69	22.74±6.16	34.4±6.39	15.34±6.53	23.24±10.46	GD+ASD, ASD> GD>CTL
**EQ** n.miss.=1	26.90±10.54	42.65±9.09	24.84±9.56	45.30±9.82	3.27±13.08	GD, CTL> GD+ASD, ASD
**CAT-Q ** n.miss.=1	121.7±25.23	88.56±27.38	119.82±25.68	73.73±23.64	92.75±32.43	ASD, GD+ASD> GD> CTL

**Conclusions:**

ASD traits may be considered clinical predictors of GD/GI symptoms.

**Disclosure of Interest:**

None Declared

## Long descriptions

### Table 1. Long description

The table consists of seven columns: Scale name, A S D (mean plus or minus s d), G D (mean plus or minus s d), G D plus A S D (mean plus or minus s d), C T L (mean plus or minus s d), Tot N equals 159 (mean plus or minus s d), and Games-Howell post-hoc comparisons (p less than 0.05).

* U G D S dash D S: A S D (39.96 plus or minus 16.17), G D (77.00 plus or minus 11.46), G D plus A S D (78.22 plus or minus 8.09), C T L (32.63 plus or minus 7.12), Tot (50.14 plus or minus 23.17). Comparison: G D, G D plus A S D greater than A S D greater than C T L.

* G S I (B U T index): A S D (0.83 plus or minus 0.84), G D (1.78 plus or minus 0.80), G D plus A S D (2.21 plus or minus 0.90), C T L (0.86 plus or minus 0.72), Tot (1.23 plus or minus 0.94). Comparison: G D, G D plus A S D greater than A S D, C T L.

* A Q: A S D (33.97 plus or minus 6.69), G D (22.74 plus or minus 6.16), G D plus A S D (34.4 plus or minus 6.39), C T L (15.34 plus or minus 6.53), Tot (23.24 plus or minus 10.46). Comparison: G D plus A S D, A S D greater than G D greater than C T L.

* E Q: A S D (26.90 plus or minus 10.54), G D (42.65 plus or minus 9.09), G D plus A S D (24.84 plus or minus 9.56), C T L (45.30 plus or minus 9.82), Tot (3.27 plus or minus 13.08). Comparison: G D, C T L greater than G D plus A S D, A S D.

* C A T dash Q: A S D (121.7 plus or minus 25.23), G D (88.56 plus or minus 27.38), G D plus A S D (119.82 plus or minus 25.68), C T L (73.73 plus or minus 23.64), Tot (92.75 plus or minus 32.43). Comparison: A S D, G D plus A S D greater than G D greater than C T L.

Navigate back to Table 1..

